# Supplementary material for: Splice-Junction-Based Mapping of Alternative Isoforms in the Human Proteome
Source: Cell Rep. Author manuscript; Available in PMC 2020 Jan 15. (PMC6961840; doi:10.1016/j.celrep.2019.11.026)
Supplement: 2 [file NIHMS1546469-supplement-2.zip › DF1/PXD006675/RightCoronaryArtery/RightCoronaryArtery_15_GTPBP2_GIGHVTDVQAITAGEAQANMGPRPPCGRPG.pdf]

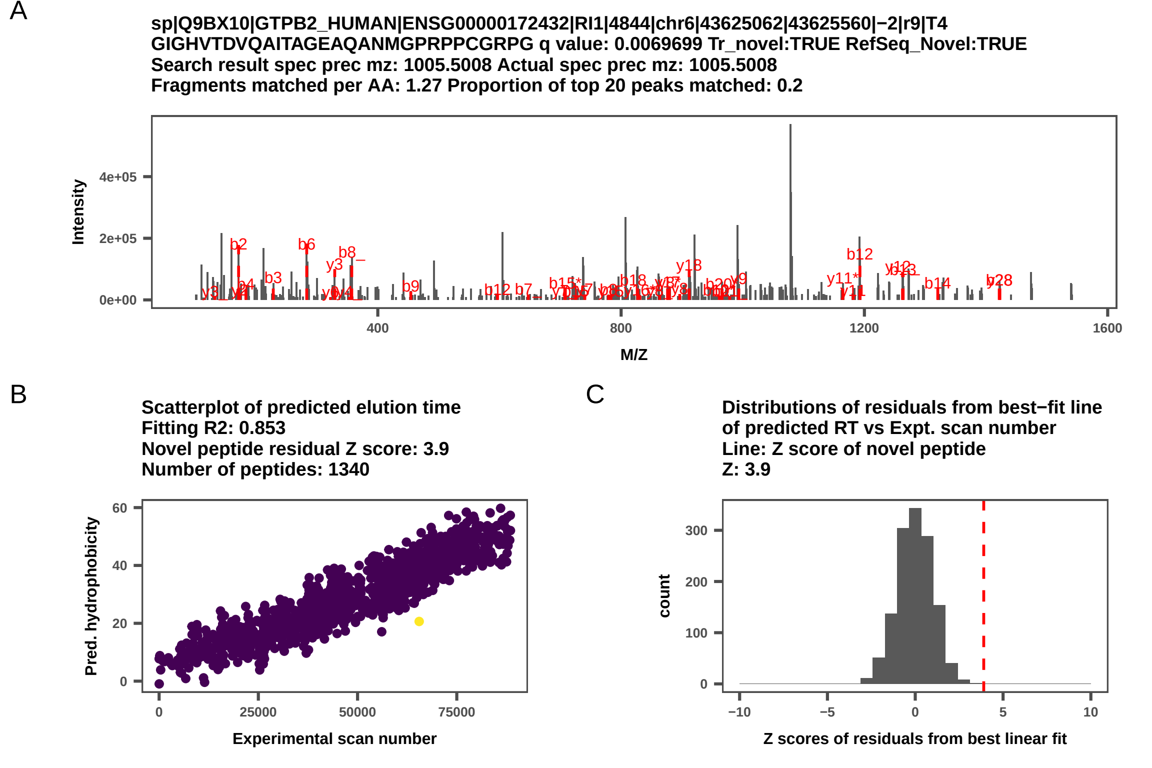
A
sp|Q9BX10|GTPB2_HUMAN|ENSG00000172432|RI1|4844|chr6|43625062|43625560|−2|r9|T4
GIGHVTDVQAITAGEAQANMGPRPPCGRPG q value: 0.0069699 Tr_novel:TRUE RefSeq_Novel:TRUE
Search result spec prec mz: 1005.5008 Actual spec prec mz: 1005.5008
Fragments matched per AA: 1.27 Proportion of top 20 peaks matched: 0.2
Intensity
4e+05
2e+05
0e+00
400
800
1200
1600
M/Z
B
Scatterplot of predicted elution time
Fitting R2: 0.853
Novel peptide residual Z score: 3.9
Number of peptides: 1340
Pred. hydrophobicity
60
40
20
0
0
25000
50000
75000
Experimental scan number
C
Distributions of residuals from best−fit line
of predicted RT vs Expt. scan number
Line: Z score of novel peptide
Z: 3.9
count
300
200
100
0
−10
−5
0
5
10
Z scores of residuals from best linear fit
